# A moderated mediation model for explaining residents’ environmental and cultural responsible behavior

**DOI:** 10.3389/fpsyg.2025.1489481

**Published:** 2025-04-16

**Authors:** Xiufang Jiang, Mollie G. Gossage

**Affiliations:** ^1^School of Law and Sociology, Xihua University, Chengdu, Sichuan, China; ^2^Chengdu Research Center for Psychosocial Services, Chengdu, Sichuan, China; ^3^Department of Anthropology, College of Letters and Science, University of Wisconsin-Madison, Madison, WI, United States

**Keywords:** environmental and cultural attitude, government trust, place attachment, relative deprivation, tourism impact, responsible behavior, sustainable development, community participation

## Abstract

**Introduction:**

The sustainable development of tourism in ethnic minority regions critically hinges on local residents’ adoption of environmentally and culturally responsible behaviors, yet the mechanisms and boundary conditions of the interplay among these core elements are unclear.

**Methods:**

This empirical study integrates the theory of planned behavior (TPB), social capital theory (SCT), place attachment theory (PAT), and relative deprivation theory (RDT) to investigate factors influencing environmentally and culturally responsible behavior (RB) among residents in western Sichuan, China. Data were collected via an online survey of residents.

**Results:**

Survey data reveal that environmental and cultural attitudes (ECA), government trust (GT), and place attachment (PA) directly promote RB, with perceived tourism impact (PTI) mediating these relationships. Additionally, relative deprivation (RD) moderates the influence of GT and PA on RB, as well as the mediating effect of PTI on the pathways from ECA and PA to RB.

**Discussion:**

This study contributes to the existing literature on tourism sustainability and destination resident behavior by illustrating that improving local responsibility requires strengthening cultural attitudes in addition to environmental attitudes, building local trust in the government, and fostering place attachment. Furthermore, the effectiveness of these goals depends on mitigating communities’ relative deprivation. These findings are significant for both theory and practice, and the conclusion contains policy insights and practical strategies for the sustainable development of ethnic minority area tourism communities.

## 1 Introduction

Rapid globalization and rampant tourism development have made sustainable development a paramount concern for tourism researchers and policymakers as well as destination management and stakeholders. Sustainability can connote a variety of domains, but ecological and cultural sustainability are particularly critical (Gkritzali, 2024; Ronström, 2024; Han, 2021; Wang et al., 2018; Qiu et al., 2018; Seraphin et al., 2018). Empirical studies help bridge the gap between lofty sustainable development goals and social, on-the-ground realities. For although environmental protection measures tend to be accepted in theory, stakeholder behavior often remains irresponsible in practice, threatening the ecological value and cultural authenticity that make destinations appealing in the first place (Ronström, 2024; Seraphin et al., 2018; Wang et al., 2018; Qiu et al., 2018). So far, research in this area has primarily focused on tourists’ environmental behavior (Han, 2021; Li et al., 2021; Liu & Wang, 2020; Qiu et al., 2018; Qiu, 2017; Cottrell, 2003), but for ethnic tourism destinations in particular, culturally responsible behavior is also a dire need. There is also a lack of research on destination residents’ environmental and cultural protection behavior compared to that of tourists, especially for residents of ethnic minority areas (Confente and Scarpi, 2021; Luo et al., 2021; He et al., 2018; Tang, 2015). This may be overlooking the people who have both the largest stake in and also greatest potential contribution for local cultural protection.

Objectively, ethnic minority areas have a wider array and variation of tangible and intangible cultural heritage. Subjectively, members of these communities are deeply invested in heritage as a marker or manifestation of their identity. Thus it is fair to say that ethnic minority areas have greater and more dire needs when it comes to cultural protection. Existing research on ecotourism in ethnic regions predominantly focuses on ecological conservation paradigms, while the cultural embeddedness within sustainable tourism development remains underexplored. This also underestimates locals’ potential contributions to multidimensional sustainability goals. Compared with those who are just passing through, people who have worked and lived at tourist destinations for long periods of time naturally become key actors for environmental and cultural protection (Jiang, 2023; He et al., 2018). By integrating cultural responsibility into environmental stewardship, this study addresses a critical oversight in tourism research, recognizing that cultural preservation is inseparable from ecological sustainability in ethnic communities. Targeting locals’ behavior, rather than tourists’, potentially reaps many more benefits with less input. Locals’ responsible behavior–for example, green, low-carbon consumption–does not only provide a model for the community itself; positive influence is literally mobilized through locals’ interactions with tourists, who then carry learned behaviors back home (Loureiro et al., 2021; Han, 2021).

This study aims to fill these gaps in the research by focusing on the environmental and cultural protection behavior and motivation of ethnic minority area residents in western Sichuan Province, Southwest China. We construct a theoretical framework that bridges theoretical divides by synthesizing the Theory of Planned Behavior (TPB), Social Capital Theory (SCT), Place Attachment Theory (PAT), and Relative Deprivation Theory (RDT)—a novel integration that captures the multidimensional drivers of responsible behavior in ethnic tourism contexts. Our model allows exploration of the backstage workings of resident behavior, with particular attention to the moderating role of relative deprivation. The contributions of this study are as follows. First, it expands the scope of existing research on environmental responsibility by incorporating the concept of cultural responsibility and highlighting the unique value of cultural factors in ethnic tourism areas. Second, this study reveals how a sense of relative deprivation moderates residents’ environmental and cultural protection activity. This provides a new perspective on the psychological dynamics residents are likely to experience in the course of tourism development. Third, it presents a new and comprehensive theoretical framework for understanding destination residents’ responsible behavior along with robust theoretical support for environmental and cultural protection policy. Finally, we include empirically based implications for management, offering practical guidance and strategies for sustainable tourism development in ethnic minority areas.

## 2 Overview of the research area

### 2.1 Study location

**Figure 1:**
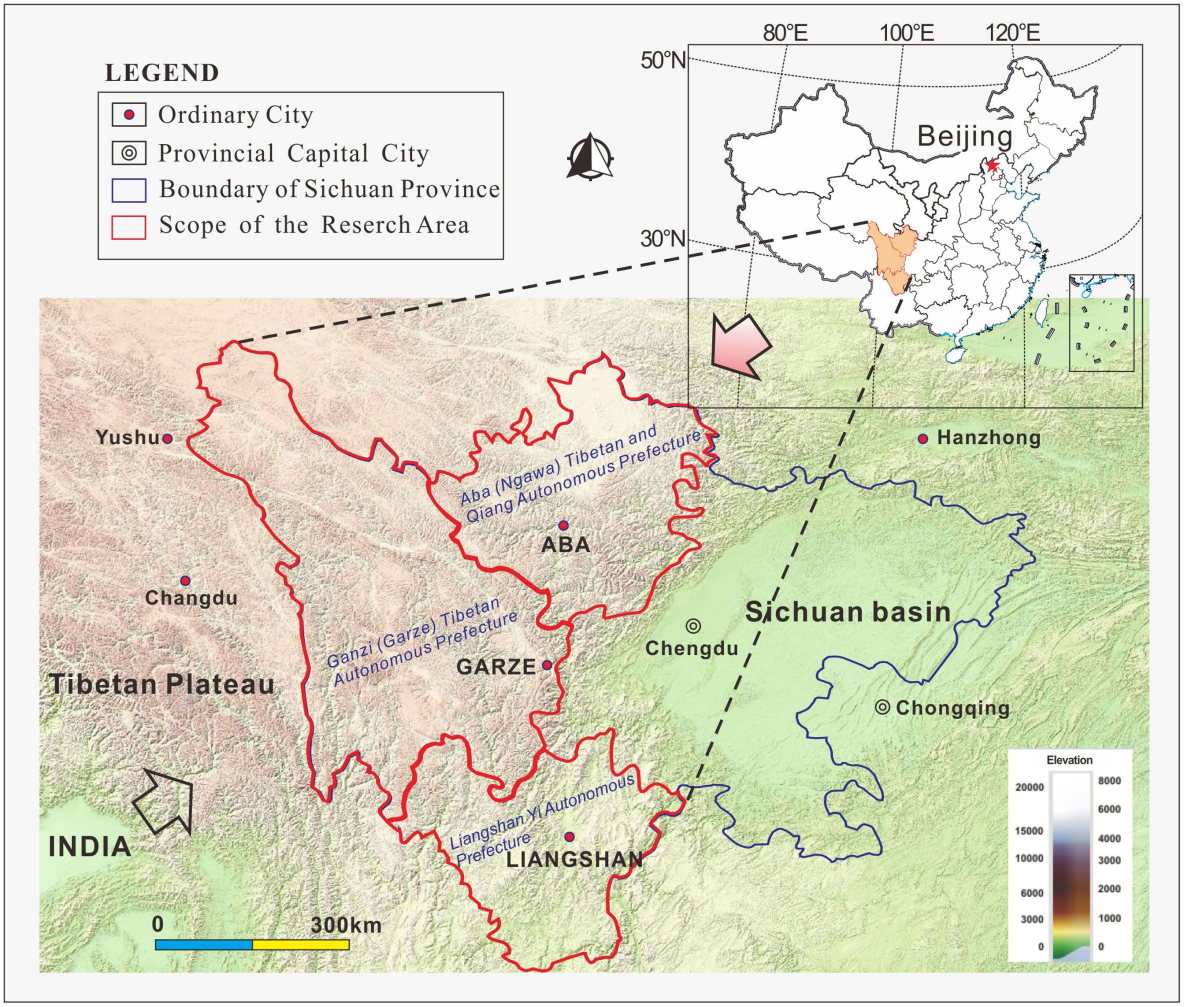
Map of the Western Sichuan Ethnic Area.

The focus of this study is the “Western Sichuan Ethnic Area,” which refers to the designated ethnic minority districts comprising western Sichuan Province in Southwest China, mainly Ganzi Tibetan Autonomous Prefecture, Aba Tibetan and Qiang Autonomous Prefecture, and Liangshan Yi Autonomous Prefecture (Figure 1). It is the most ethnically diverse region of Sichuan, with the largest concentrations of ethnic minorities, incorporating the majority of Kham (Kang)—the second-largest Tibetan cultural area in China—as well as the largest concentration of Yi in China and the remaining extent of the Qiang homeland. The diversity of culture in this area is rivaled only by the diversity of biomes. Highly variable elevation and climatic conditions foster a unique and wide array of flora, fauna, and landscapes, while north–south mountain ranges and river valleys form a key ecological corridor connecting not only different regions of China, but East Asia and South Asia on the whole. Western Sichuan’s influence on regional and global ecology is undeniable.

### 2.2 Ecological and cultural value

The Western Sichuan Ethnic Area is of great significance in the context of China’s ecological and cultural landscape. In China, “National Key Ecological Function Zone” (NKEFZ) is used to designate an area that is sufficiently ecologically rich or fragile to warrant certain restrictions on its use. Intensive, large-scale development is curbed within these zones to achieve balanced, sustainable, and optimized development at the national scale. The Western Sichuan Ethnic Area is not only an important NKEFZ on its own; within its boundaries are numerous nationally managed and internationally recognized nature reserves, including National Geoparks and Natural World Heritage sites. These reserves supply precious resources for biodiversity conservation, scientific research–and cultural heritage. With its dramatic mountain ranges, deep river valleys, and high plateau grasslands, western Sichuan’s geography has fostered an array of unique lifestyles and highly localized traditions. At the same time, as part of an ancient borderland and migration corridor, it is a place where multiple languages, religions, artistic traditions, and ethnic communities have engaged in long-term exchange or amalgamation. Rich biodiversity and rare cultural assemblages exist side-by-side, which has drawn substantial international attention to this area.

Areas of ethnic minority concentration, whose residents maintain distinct rural identities, have long been a focus of heritage and tourism resource development, as well as critical subjects in the research on sustainable ethnic tourism (Liu, 2023). Tourism types are generally defined from the perspective of tourist desires, and ethnic tourism is no exception. Zhang et al. (2017) adapt and expand Valene L. Smith’s definition (Smith 1989, p. 4) to China’s socio-cultural context and scholarly discourses, stating: “Ethnic tourism refers to a process wherein tourists fulfill their aesthetic demands through active participation, observation, and immersion in the unique cultural practices or lifestyles of specific ethnic groups… The essence of ethnic tourism inherently manifests as interethnic communication or cross-cultural observation and experience.” The motivating force behind ethnic tourism development is an interest in different ways of life, but an influx of tourists and outside businesses tends to be homogenizing or dislocating for local cultures. This underscores the importance of purposeful heritage protection, a win-win for ethnic tourists and ethnic tourism area residents alike.

### 2.3 Research value and implications

The above should make evident the academic and pragmatic reasons for selecting the Western Sichuan Ethnic Area for a study on environmentally and culturally responsible behavior. Western Sichuan’s ethnic minority regions have not only rich cultural heritage and ecological importance, but strategic significance for national stability, ethnic relations, and ecological security. At the same time, the area confronts a multitude of challenges that directly influence residents’ environmental and cultural responsible behavior. Communities here continue to grapple with economic underdevelopment and a substantial lag in per capita GDP when compared to global, national, and even provincial benchmarks. This economic disparity, compounded by the fragile ecosystems of the region and the necessity for strict adherence to ecological protection measures, presents a unique array of difficulties. Residents often find themselves in a delicate balance between their traditional livelihoods, which may heavily rely on natural resource extraction, and the imperatives of sustainable development and environmental preservation. Furthermore, limited infrastructure, and the sheer distance between communities and from developed centers, hinder access to education and modern facilities, further complicating efforts to foster environmental and cultural responsibility. It is crucial to understand and address these entrenched problems if we are to cultivate a sense of environmental and cultural stewardship among area residents.

## 3 Theoretical foundation and study hypotheses

The study hypotheses build upon four key theories: the theory of planned behavior (TPB), social capital theory (SCT), place attachment theory (PAT), and relative deprivation theory (RDT). We understand these four theories as interrelated to form a comprehensive explanatory framework for the responsible behavior of residents in ethnic tourism areas. The TPB provides the foundational motivational factor through environmental and cultural attitudes (ECA), which directly influence residents’ behavior. The SCT supplements this framework by introducing government trust (GT) as a key variable, highlighting the positive impact of social relationships and trust on behavior. The PAT further explains how place attachment (PA) translates residents’ emotional connections to their local area into protective actions. The RDT provides a theoretical framework for understanding how relative deprivation (RD) felt by residents in social comparisons might influence their responsible behavior. Specifically, RD could potentially moderate the effects of environmental and cultural attitude (ECA), government trust (GT), and place attachment (PA) on responsible behavior, as well as influence the mediating role of perceived tourism impact (PTI). This framework allows us to explore the complex interplay between psychological states and environmental attitudes in shaping responsible behavior among residents in ethnic tourism areas. These theories complement each other, providing a multidimensional explanation of the formation mechanisms of responsible behavior among residents in ethnic tourism areas from the perspectives of attitudes, social trust, emotional attachment, and psychological states. The following subsections clarify interrelations between these foundational theories and the hypotheses of this study.

### 3.1 Environmental and cultural attitude and responsible behavior

Attitude is an important predictor of behavior. According to the TPB, there are three factors that determine an individual’s intention to act: attitude, subjective norms, and perceived behavioral controls. Environmental attitude (EA) refers to the extent that one recognizes and supports solutions to environmental problems, or one’s personal willingness to contribute to potential solutions (Dunlap et al., 2000). Some scholars express a similar concept with terms such as environmental “concern” (Li et al., 2021) or “values” (Stern et al., 1999). Our study takes this idea further by incorporating cultural heritage and protection, therefore employing the term “environmental and cultural attitude” (ECA). ECA measures the degree to which one is aware of and supports solutions to threats against environmental and cultural heritage, or one’s personal willingness to contribute to potential solutions in these areas. So far, a number of studies have found that EA is the largest influencer or most reliable indicator of environmentally responsible behavior (Tanner and Wölfing Kast, 2003; Wang et al., 2023; Williams et al., 2015). Liu and Wang (2020) found high correlation between tourists’ environmental attitudes and environmentally responsible behavior, while Wang et al. (2019) found moderate correlation. Cottrell (2003) observed a weak yet positive correlation between tourists’ environmental attitudes and responsible behavior, similar to findings by Kurz et al. (2007) and Bamberg (2003). In light of the existing work, we put forth the following hypothesis:

H1: Environmental and cultural attitude has a positive impact on responsible behavior.

### 3.2 Government trust and responsible behavior

SCT reveals the impact of social relationships–within which trust, emotion, and norms are embedded–on attitudes and behaviors of the individual or the group, as well as their role in advancing individual or group interests (Portes, 2000). At the collective level, social capital manifests emotionally as mutual trust and confidence. Trusting relations are the bedrock of group cohesion (Brunie, 2009), which facilitates a community’s problem-solving and general satisfaction (Portes and Landolt, 1996; Dallago et al., 2009). Social capital is also an important factor shaping individual behavior (Orgaz-Agüera et al., 2022; Liu et al., 2014). A study by Liu et al. (2014) used SCT to investigate the relationship between social capital and environmentally responsible behavior among residents of an ecotourism zone. Their findings demonstrate a positive correlation between the social capital reserve and residents’ environmental responsibility.

Trust is considered the most crucial element of social capital. This includes both trust among residents and trust in the authorities (Purdue, 2001). When trust in authorities is high, it is easier to launch collective actions and enhance residents’ senses of security, belonging, and identity. In short, trust provides a basis for social order (Jiang et al., 2022; Putnam et al., 1993). As a key component of social capital, “government trust” (GT) refers to one’s confidence that the government is able to govern–both itself and others–and one’s satisfaction with the government’s institutional arrangements Lu and Li (2021). For a tourist destination, high GT indicates residents believe that the government is carrying out tourism policy and development in a fair and impartial manner. High GT stimulates residents’ sense of belonging, which in turn improves their perceptions of tourism impacts and their responsible behavior (Jiang, 2023). A study on ecotourism by Ramón-Hidalgo and Harris (2018) emphasizes that social capital and political empowerment are both critical factors for successful community-based natural resource management. It may be inferred that as GT increases, tourist destination residents are more likely to espouse enthusiasm and support for tourism development, which in turn leads to more environmentally responsible behavior. Trust, therefore, not only promotes cooperation within local communities; it also provides a foundation for effective government-community collaboration, creating a path for the joint advancement of sustainable development. In light of the above theory and research, we propose the following hypothesis:

H2: Government trust has a positive impact on responsible behavior.

### 3.3 Place attachment and responsible behavior

Place Attachment Theory (PAT) explains how emotional bonds to local landscapes and traditions (e.g., Tibetan mountain worship rituals) translate into protective actions. Specifically, PAT posits that residents with stronger place attachment perceive themselves as custodians of both natural and cultural heritage (Buta et al., 2014). PAT offers a powerful framework for understanding how emotional bonds translate into a place-oriented sense of responsibility and protective behaviors. Research indicates that place attachment (PA) goes beyond mere emotional connection; it becomes a motivating force that compels a person to act in an environmentally responsible way (Qiu, 2017; Wan et al., 2014; Fan et al., 2014). In tourist studies, PA is found to have a significant positive impact on tourists’ environmentally responsible behavior (Wu et al., 2023; Buta et al., 2014). A study by Li et al. (2021) refines this analysis, demonstrating that strong PA significantly promotes both tourists’ passive compliance with environmental regulations and their active participation in pro-environmental activities. Tourist PA is thus a relevant, operationalizable emotion for environmental and green tourism initiatives.

PAT suggests that these relationships are scalable–in other words, the stronger an individual’s attachment to a given place, the stronger their sense of responsibility, and the more inclined they are to target their environmentally protective actions toward that place. There is wide empirical support for PAT. For example, Buta et al. (2014) found that PA mediates relations between community attachment and environmentally responsible behavior, and similar studies have shown that PA mediates the relations between psychological ownership of a destination (Zhang and Zeng, 2019) or place image (Shen et al., 2019) and environmentally responsible behavior. Evidently, emotional ties to place not only stimulate a direct motivation to conserve; they also instill a sense of responsibility manifesting across a range of practical activities. This underscores the importance of PA as a psychological mechanism for environmentally responsible behavior. Accordingly, we propose the following hypothesis:

H3: Place attachment has a positive impact on responsible behavior.

### 3.4 The mediating effect of perceived tourism impact

The effects of tourism development on a destination’s economy, society, culture, and ecology are undeniably a mixed bag (de Sousa et al., 2017; Sinclair-Maragh et al., 2015; Salerno et al., 2013). However, beyond positive or negative changes from tourism itself, residents’ positive or negative perceptions and attitudes have real economic, sociocultural, and ecological effects. If locals embrace tourism development, it can make a destination more attractive, whereas pessimism, aversion, and unwelcoming behavior toward tourists may influence destination image and pose threats to tourism sustainability and even safety. Residents’ perceived tourism impact (PTI) is generally separated along three lines of economic, sociocultural, and environmental impacts (Kim et al., 2021; Rasoolimanesh et al., 2017; Stylidis et al., 2014), though some studies distinguish additional dimensions including the institutional (Cottrell et al., 2013; Cottrell and Cutumisu, 2006), relative deprivation (Luo et al., 2021), and agrarian livelihood (Xu and Sun, 2020).

Slabbert et al. (2020) confirm tourism impact as a predictive factor for residents’ opinions and interest levels in tourism activities. In their study, the tourism industry benefited destination development as well as local prosperity, fostering positive community attitudes. Beyond these effects, Jiang (2023) found that PTI mediates the effects of place image, social capital, relative sense of gain, government trust, and tourism involvement on destination resident quality of life. Lu and Li (2021) likewise report that PTI mediates the effect of government trust on resident quality of life and supportive attitudes. Meanwhile, Pang et al. (2018) found that PTI mediates the effects of place attachment and community involvement in resource conservation behavior. Clearly, if we are to understand the interplay between resident psychology and sustainable tourism development, we must attend to the multiple effect pathways of PTI. Based on the established findings, we propose the following hypotheses:

H4: Environmental and cultural attitude indirectly influences responsible behavior through perceived tourism impact.

H5: Government trust indirectly influences responsible behavior through perceived tourism impact.

H6: Place attachment indirectly influences responsible behavior through perceived tourism impact.

### 3.5 The moderating effect of relative deprivation

Individuals’ psychology and ECA do not exist in static isolation. Subjective experiences and relational perceptions also have marked effects on one’s responsible behavior, including environmental behavior and attitudes (Wu et al., 2023). In the context of tourism development, the substantial gains and losses at stake make social comparison a particularly salient factor.

In behavioral psychology, equity theory suggests that individuals perceive equity, or fairness, by comparing their own returns on investments with others’. When such comparison identifies disparity, it can result in a sense of either gain or deprivation. This is important because one’s sense of relative gain serves as a shorthand measure for one’s social standing, which is integral to an overall positive emotional experience (Ye et al., 2018). Feeling that one has advantages in society has direct implications for one’s happiness and quality of life (Jiang, 2023).

On the other hand, a sense of relative deprivation (RD) arises when one seems to have lost out, or benefitted less, compared with people in other groups or with one’s past self (Peng and Wang, 2012; Luo et al., 2021). RD psychology is common among tourist destination residents. Whenever the distribution of tourism development benefits is uneven, or there is a decline in some area of life following tourism development, a sense of loss naturally arises (Luo et al., 2021; Wang, 2015). Research indicates that destination communities with a strong sense of RD are more negative about tourism, which can hinder local sustainable development (Luo et al., 2021; Xu and Sun, 2020). Considering the documented role of RD in moderating environmental attitudes and responsible behavior, we put forth the following hypotheses:

H7: Relative deprivation moderates the effect of environmental and cultural attitude on responsible behavior.

H8: Relative deprivation moderates the effect of government trust on responsible behavior.

H9: Relative deprivation moderates the effect of place attachment on responsible behavior.

Inferring from hypotheses 4 through 9, it is clear that perceived tourism impact’s mediation may be moderated by relative deprivation; therefore, we propose the following additional hypotheses:

H10: Relative deprivation moderates the mediating effect of perceived tourism impact in the relationship between environmental and cultural attitude and responsible behavior.

H11: Relative deprivation moderates the mediating effect of perceived tourism impact in the relationship between government trust and responsible behavior.

H12: Relative deprivation moderates the mediating effect of perceived tourism impact in the relationship between place attachment and responsible behavior.

**Figure 2:**
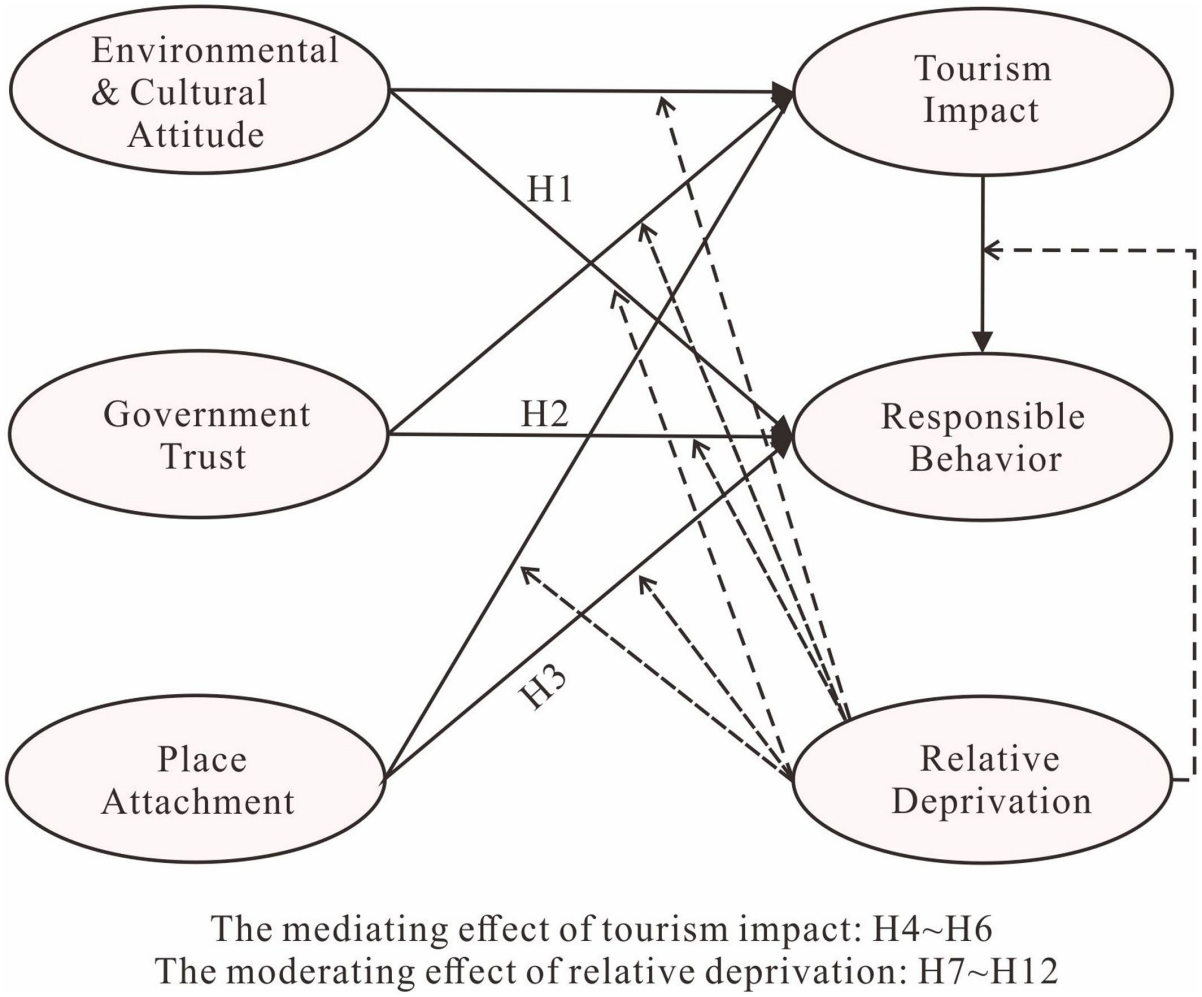
Conceptual model of the study hypotheses.

The conceptual diagram below (Figure 2) is based on the above set of research hypotheses.

## 4 Study design and data collection

### 4.1 Questionnaire design and variable measurement

To ensure reliability and validity of the data, questionnaire design elements were adapted from well-established scales to fit the purpose and context of this study. The main body of the questionnaire uses a 5-point Likert scale, where 1 indicates “strongly disagree” and 5 indicates “strongly agree.”

Drawing on questionnaires by Tang (2015) and Pang et al. (2018), we measured environmental and cultural attitude with five questionnaire items. These items were selected because they comprehensively cover residents’ attitudes toward the value of local culture and environment and their willingness to protect them. Specifically, Tang (2015) emphasized the intrinsic value of local culture and residents’ responsibility to protect it, while Pang et al. (2018) highlighted the importance of protecting culture and environment for sustainable development. By integrating these perspectives, we aimed to capture a holistic view of residents’ attitudes. The items include: “Local culture is valuable and should be protected;” “The local natural environment is valuable and should be protected;” “A portion of local tourism revenue should be used for cultural and environmental protection;” “Local culture and environment should be protected both now and in the future;” “I have a responsibility to actively participate in the protection of culture and environment.” Cronbach’s alpha for these items was 0.96, indicating excellent reliability.

Government trust is measured by four questionnaire items adapted from the classic questionnaire by Nunkoo (2015). These items were chosen because they effectively capture residents’ perceptions of the government’s role and policies in tourism development, which is crucial for understanding residents’ willingness to engage in environmental and cultural protection activities. The items include: “The government has a long-term plan for tourism development;” “The government has policies that encourage and support locals’ participation in tourism development;” “The government pays attention to residents’ desires and demands in tourism development;” “In the course of tourism development, the government considers residents’ interests.” Cronbach’s alpha was 0.90, indicating excellent reliability.

Measurement of place attachment draws on Tang (2015) and Pang et al. (2018). Tang (2015) used six items to measure place attachment, such as “Living here makes me more satisfied than living in other places” and “I would be very sad if I had to leave here.” These items effectively reflect residents’ emotional attachment and sense of belonging to the local area. Pang et al. (2018) further emphasized the importance of place attachment in residents’ community participation, suggesting that it reflects residents’ shared responsibility for community development and their enjoyment of its outcomes. Based on these studies, we selected the following five items to measure place attachment: “I love local culture;” “I will actively safeguard local culture;” “I am more satisfied living here than in other places;” “I think that here is an ideal place to live;” “I am proud and honored to be living in this place.” For these items, Cronbach’s alpha was 0.96, indicating excellent reliability.

Derived from Pang et al. (2018), responsible behavior is measured by three questionnaire items. These items were selected because they effectively capture proactive behaviors that residents may exhibit in protecting the local environment and cultural heritage. The items include: “I will actively comply with all environmental and cultural protection management measures;” “I will actively protect local ecological environment and cultural heritage;” “I will actively prevent others from damaging or destroying the local natural environment and cultural heritage.” Cronbach’s alpha for these items was 0.96, indicating excellent reliability.

Our design for perceived tourism impact measurement first draws on the research of Ap and Crompton (1998), which uses a scale structure to evaluate respondents’ attitudes toward ecotourism more effectively. Like Ap and Crompton, we measured two components of PTI–“belief,” by asking about the extent of local change brought by tourism, and “evaluation,” by asking respondents to rate their level of like or dislike for the given change. In our study, respondents rated belief statements on a 5-point scale (1 for a large decrease, 5 for a large increase) and evaluation statements on a 4-point scale (1 for dislike, 4 for like). Belief and evaluation ratings were multiplied, with possible products ranging from 1 to 20. A high value indicates strong support for ecotourism, while a low value indicates a negative attitude toward ecotourism.

Second, with reference to Jia et al. (2021), we created nine PTI measurement items: “wildlife (plants, birds, and animals);” “environmental quality (air, water, etc.);” “opportunities to restore and protect historic architecture;” “understanding and awareness of cultural heritage;” “contact with tourists and their culture;” “price of local goods and services;” “individual income;” “employment opportunities;” and “government revenue.” Cronbach’s alpha was 0.82, indicating good reliability.

The measurement method for relative deprivation is adapted from Luo et al. (2021), with four questionnaire items: “Compared to other locals, how would you rate your economic situation?”; “Compared to other locals, how would you rate your sociocultural standing?”; “Compared to other locals, how would you rate your environmental health?”; “Compared to other locals, how would you rate the quality of your institutions?.” Respondents selected values 1 through 5 for each item, with 1 indicating “well below average” and 5 indicating “well above average.” Cronbach’s alpha for these items was 0.86, indicating good reliability.

Demographic variables collected with this questionnaire include gender, age, educational attainment, ethnicity, place of origin, place of residence (and distance from core tourism areas), duration of residence, occupation, source of household income, and annual earnings from tourism sources.

### 4.2 Sample survey data

The design and distribution phase for this study extended from January 2022 to July 2024. Questionnaires were distributed to Western Sichuan Ethnic Area residents via the online tool “Questionnaire Star” (*Wenjuanxing*). Proportionate stratified random sampling ensured that representation in each stratum–gender, age, level of education–was proportional to its share within the whole population. We did not gather questionnaire responses from multiple adults per household.

The Sichuan Province Statistical Yearbook for 2020 records that Ganzi, Aba, and Liangshan Prefectures had 6.79 million permanent residents at year’s end. According to Wu’s (2009) sample size formula, the smallest acceptable sample for this size population would be 385 people. The effective sample size for this study is 402, which exceeded the minimum threshold for robust statistical inference, thereby enhancing the generalizability of the findings. Among this sample, men comprise 57.71% and women 42.29% of the total; 13.18% are 18 to 24 years old; 28.61% are 25 to 34; 25.87% are 35 to 44; and 27.11% are 45 to 54. Over 60% of respondents have a junior-high level of education. In terms of ethnicity, 22.64% are Han; 29.85% are Tibetan; 25.87% are Yi; 20.4% are Qiang. The length of local residence is mainly concentrated in two categories–6 to 10 years (27.86%) and 11 to 20 years (22.64%). Just over half (51.99%) of study participants are not directly involved in the tourism industry, though slightly more (55.22%) confirmed that their household income is affected by tourism. Those who are directly involved in tourism mostly engage in frontline service work. Annual income from tourism sources varies substantially, with highest concentrations in the “30,001–50,000” and the “less than 5,000 RMB” categories, accounting for 36.07 and 31.59% of respondents, respectively (Table 1).

**Table 1:** Background characteristics of participants.

Variable	Subgroup	Percentage (%)
Gender	Male	57.71
	Female	42.29
Age	18–24	13.18
	25–34	28.61
	35–44	25.87
	45–54	27.11
	55+	5.22
Educational level	Primary school or below	7.46
	Junior High School	30.6
	Senior High School/Vocational	30.1
	Bachelor’s/Associate	25.62
	Postgraduate	6.22
Ethnicity	Han	22.64
	Tibetan	29.85
	Qiang	20.4
	Yi	25.87
	Other ethnic groups	1.24
Duration of local residence	5 years or less	18.41
	6 ~ 10 years	27.86
	11 ~ 20 years	22.64
	21 ~ 30 years	16.92
	Over 30 years	14.18
Role in tourism development	Not directly involved in tourism	51.99
	Tourism operator	42.54
	Tourism manager	5.47
Personal annual tourism income	5,000 yuan or less	31.59
	5,001 ~ 10,000 yuan	17.41
	10,001 ~ 30,000 yuan	8.21
	30,001 ~ 50,000 yuan	36.07
	Over 50,000 yuan	6.72
Family income sources	Mainly from tourism	14.68
	Partly from tourism	31.09
	Marginally from tourism	9.45
	Not from tourism	44.78

### 4.3 Research methods

This study used online analytics software SPSSAU for tests of reliability and validity, descriptive statistics, and correlation coefficients. From there, we used linear regression analysis to investigate the effects of ECA, GT, PA, PTI, and RD on responsible behavior. Next, we employed bootstrapping to assess the mediating effect of perceived tourism impact, and moderating effect analysis to verify the role of RD. Finally, we used model 59 for moderated mediation analysis to ascertain whether the mediating effect of perceived tourism impact is moderated by relative deprivation.

## 5 Data analysis

### 5.1 Multicollinearity, autocorrelation, and normality tests

When linear regression analysis was applied to test for collinearity and autocorrelation, our model passed the *F*-test (*F* = 361.132, *p* < 0.05), indicating statistical significance. The maximum variance inflation factor (VIF) was 3.56; because all VIF values were below 5, there is no covariance issue. The Durban-Watson (DW) statistic was 1.56, demonstrating there is no correlation between the model and sample data. All together, these results attest to the quality of the model. Meanwhile, all measurement items were found to be significant (p < 0.05) through a Kolmogorov–Smirnov test for normal distribution. Absolute value of the skewness coefficient ranged from 0.02 to 0.86, while absolute value of the kurtosis coefficient ranged from 0.01 to 2.23–well below the critical values of 3 and 8, respectively; our sample data passed the normality test.

### 5.2 Descriptive statistical analysis

Pearson correlation analysis was used to measure associative relationships between variables. Findings, as shown in Table 2, demonstrate that ECA, GT, PA, PTI, and RD correlate significantly with responsible behavior, providing rationale for further hypothesis testing.

**Table 2:** Means, standards of deviation, and correlation coefficients.

Variable	Mean	Std Dev	1	2	3	4	5	6
1. Responsible behavior	4.1	0.65	1					
2. Environmental and cultural attitude	4.11	0.65	0.88***	1				
3. Government trust	3.88	0.68	0.68***	0.68***	1			
4. Place attachment	4.02	0.65	0.76***	0.76***	0.56***	1		
5. Perceived tourism impact	12.53	3.62	0.76***	0.76***	0.67***	0.76***	1	
6. Relative deprivation	3.17	0.68	0.74***	0.75***	0.68***	0.66***	0.78***	1

*** *p* < 0.001.

### 5.3 Hypothesis testing

#### 5.3.1 Direct effects

Regression analysis was used to test the direct effects of five variables–ECA, GT, PA, PTI, and RD–on responsible behavior (RB). The resulting R-squared was 0.82, meaning that these variables can account for 82% of variation in RB. More specifically, the regression coefficients were 0.581 (*t* = 14.571, *p* < 0.01) for ECA, 0.076 (*t* = 2.537, *p* < 0.05) for GT, 0.153 (*t* = 4.36, *p* < 0.01) for PA, and 0.028 (*t* = 3.38, *p* < 0.01) for PTI. This verifies that each of these four have a significant and positive impact on responsible behavior, which validates study hypotheses 1 through 3. The regression coefficient for RD, however, was 0.041 (*t* = 1.103, *p* > 0.05), indicating that relative deprivation does not have a significant direct effect on responsible behavior.

Among the above-mentioned significant positive factors, ECA had the greatest impact (*β* = 0.58, p < 0.01). This means that for every unit increase in environmental and cultural attitude, the expected increase in RB is statistically significant. In comparison, GT and PA are less influential (*β* = 0.08, *p* < 0.05 and *β* = 0.15, *p* < 0.01, respectively). Clearly, locals’ positive attitudes towards environmental and cultural protection, as well as their trust and commitment to the government and community, are key factors motivating them to behave more responsibly.

#### 5.3.2 Mediation effects

Bootstrap sampling was used to test for mediation. For robust results, five thousand iterations were conducted. Results, as shown in Table 3, show that PTI partially mediates the effects of ECA, GT, and PA on RB. Path a (the effect of the independent variable on the mediating variable), path b (the effect of the mediating variable on the dependent variable), and path c′ (the direct effect of the independent variable after controlling for the mediating variable) are all significant.

**Table 3:** Mediating effects.

	c total effect	a	b	a*b mediating effect	a*b (95% BootCI)	c′ direct effect	Conclusion	Effect size
ECA → PTI → RB	0.64***	1.61***	0.02*	0.03	0.00–0.06	0.61***	Partial mediation	4.25%
GT → PTI → RB	0.12***	1.36***	0.02*	0.02	0.00–0.05	0.10**	Partial mediation	18.70%
PA → PTI → RB	0.21***	2.22***	0.02*	0.04	0.00–0.07	0.18***	Partial mediation	17.45%

***p < 0.001, **p < 0.01, *p < 0.05.

ECA’s mediation accounts for 4.25% of the total effect, indicating that environmental and cultural attitudes indirectly promote responsible behavior by improving the perception of tourism impact. Likewise, it can be seen that government trust has not only a direct but also a mediating effect on RB through PTI; GT’s mediating effect accounts for 18.7% of the total. Finally, with a mediating effect size 17.45% of the total, place attachment was also confirmed to indirectly promote responsible behavior through PTI. Mediation test results validate study hypotheses 4 and 6 while emphasizing the overall importance of perceived tourism impact in promoting responsible behavior.

#### 5.3.3 Moderation effects

**Figure 3:**
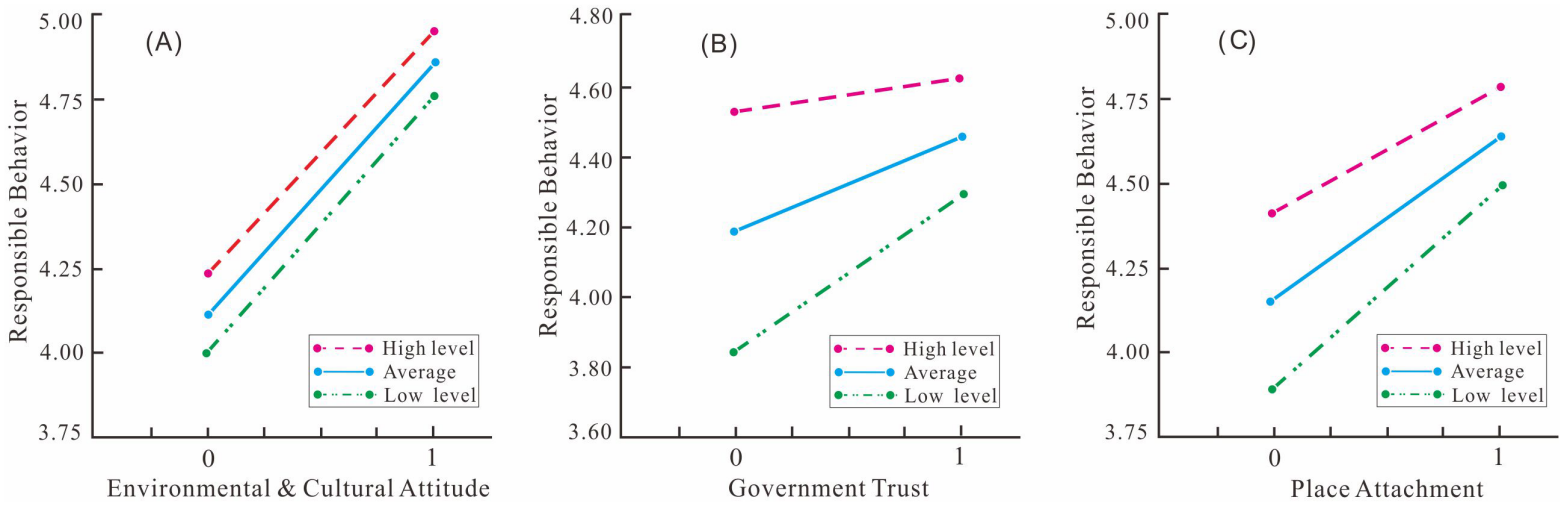
The moderating effects of relative deprivation.

Moderating effect analysis revealed that RD significantly moderates the respective relationships of ECA, GT, and PA with responsible behavior, but found no statistically significant interaction term between ECA and RD (*t* = −1.089, *p* = 0.277 > 0.05). This implies that the positive influence ECA exerts on responsible behavior is not affected by changes in relative deprivation (Figure 3A). Therefore, environmental and cultural attitude consistently promotes responsible behavior regardless of the level of relative deprivation; study hypothesis 7 is not supported.

In contrast, the interaction term between GT and RD is statistically significant (*t* = −7.790, *p* < 0.05). Figure 3B shows that government trust has a more pronounced positive effect when the relative deprivation is low. This suggests that residents are more likely to engage in responsible behavior out of their trust in the government when they feel less deprived. However, GT’s positive effect diminishes at higher levels of relative deprivation. A strong sense of comparative loss thwarts the positive influence of government trust on responsible behavior. Study hypothesis 8 is supported by this result.

The interaction term between PA and RD is likewise significant (*t* = −5.174, *p* < 0.05). As demonstrated by Figure 3C, when relative deprivation is low, place attachment has a more marked effect on residents’ responsible behavior, indicating that a strong attachment to place can effectively promote responsible behavior as long as residents do not also feel deprived. If the sense of deprivation is high, the positive effect from one’s ties to place weakens. Therefore, even when residents have deep place attachment, if they also feel a strong sense of deprivation, they may decline to engage in responsible behavior. Study hypothesis 9 is validated by this result.

#### 5.3.4 Conditional indirect effects

Moderated mediation tests were run using model 59 of the SPSS macro PROCESS (which assumes all three mediation paths are moderated). The goal was to see if varying degrees of the moderating variable, relative deprivation, led to differences in the mediating role of perceived tourism impact. The results are shown in Table 4. We found that as ECA indirectly influences responsible behavior, the mediating effect of PTI is significantly impacted by the level of RD. When RD was at low or mean levels, the mediating effect was significant–with respective effect values of 0.14 and 0.07, respectively (boot 95% CI excluding 0). In other words, at low or moderate levels of relative deprivation, positive tourism perceptions successfully strengthen the positive link between environmental and cultural attitude on responsible behavior. However, when RD was high, its mediating effect ceased to be significant (boot 95% CI including 0). This means that under conditions of high relative deprivation, strong PTI fails to strengthen the effect of ECA on responsible behavior. Study hypothesis 10 is supported by these results.

**Table 4:** Conditional indirect effects.

Independent variable	Moderating variable level	Level value	Effect	BootSE	BootLLCI	BootULCI
Environmental and cultural attitude	Low (–1 SD)	2.48	0.14	0.07	0.00	0.27
	Mean	3.17	0.07	0.02	0.03	0.12
	High (+1 SD)	3.85	0.02	0.04	−0.03	0.12
Government trust	Low (–1 SD)	2.48	0.08	0.03	0.03	0.17
	Mean	3.17	0.08	0.03	0.04	0.14
	High (+1 SD)	3.85	0.07	0.03	0.02	0.15
Place attachment	Low (–1 SD)	2.48	0.14	0.04	0.06	0.23
	Mean	3.17	0.09	0.03	0.03	0.15
	High (+1 SD)	3.85	0.05	0.03	−0.01	0.11

Unlike ECA, when GT indirectly influences responsible behavior, the mediation effect of PTI is significant across variable levels of relative deprivation. Effect values were 0.08 at low RD, 0.08 at mean RD, and 0.07 at high RD (boot 95% CI excluding 0). Therefore, the positive effect that government trust indirectly exerts on responsible behavior through tourism impact remains consistent across all levels of relative deprivation. With no significant moderated mediation; study hypothesis 11 is not supported.

The pattern for place attachment was similar to that for ECA. When PA indirectly influenced responsible behavior, the mediating effect of PTI was significant only at low and mean levels of RD, with effect values of 0.14 and 0.09, respectively (boot 95% CI excluding 0). Evidently, place attachment can promote responsible behavior by enhancing perceptions of tourism under these conditions. However, when RD was at a high level, the mediating effect became insignificant (boot 95% CI including 0). This means that when residents feel highly deprived, it attenuates the indirect positive effect of place attachment on responsible behavior via PTI. Study hypothesis 12 is proven valid.

### 5.4 Differential analysis

The study employed t-tests and analysis of variance (ANOVA) to examine whether significant differences existed in residents’ responsible behavior across various demographic and socio-economic backgrounds, including gender, age, education level, ethnicity, length of residence, role in tourism development, personal annual tourism income, and family income sources (Table 5).

**Table 5:** Difference analysis on varied demographic characteristics in residents’ responsible behavior.

Variable	Subgroup	Mean Score ± SD	Statistical Significance
Gender	Male	3.96 ± 0.72	*t* = −5.62, *p* < 0.001
	Female	4.30 ± 0.48	
Age	18–24	4.11 ± 0.67	*F* = 20.94, *p* < 0.001
	25–34	4.22 ± 0.62	
	35–44	4.24 ± 0.48	
	45–54	4.06 ± 0.67	
	55+	3.00 ± 0.00	
Educational level	Primary School or Below	4.20 ± 0.53	*F* = 19.7, *p* < 0.001
	Junior High School	4.20 ± 0.54	
	Senior High School/Vocational	3.71 ± 0.77	
	Bachelor’s/Associate	4.37 ± 0.46	
	Postgraduate	4.29 ± 0.38	
Ethnicity	Han	4.29 ± 0.46	*F* = 19.12, *p* < 0.001
	Tibetan	4.32 ± 0.70	
	Qiang	3.66 ± 0.65	
	Yi	4.01 ± 0.51	
	Other ethnic groups	4.60 ± 0.89	
Duration of local residence	5 years or less	4.11 ± 0.45	*F* = 38.94, *p* < 0.001
	6 ~ 10 years	4.15 ± 0.38	
	11 ~ 20 years	3.53 ± 0.67	
	21 ~ 30 years	4.44 ± 0.70	
	Over 30 years	4.52 ± 0.56	
Role in tourism development	Not directly involved in tourism	3.84 ± 0.66	*F* = 44.91, *p* < 0.001
	Tourism operator	4.40 ± 0.46	
	Tourism manager	4.29 ± 0.77	
Personal annual tourism income	5,000 yuan or less	4.15 ± 0.46	*F* = 26.39, *p* < 0.001
	5,001 ~ 10,000 yuan	4.43 ± 0.54	
	10,001 ~ 30,000 yuan	4.44 ± 0.67	
	30,001 ~ 50,000 yuan	3.74 ± 0.66	
	Over 50,000 yuan	4.54 ± 0.56	
Family income sources	Mainly from tourism	4.42 ± 0.59	*F* = 14.05, *p* < 0.001
	Partly from tourism	4.25 ± 0.41	
	Marginally from tourism	4.09 ± 0.43	
	Not from tourism	3.90 ± 0.76	

#### 5.4.1 Gender

A t-test revealed a statistically significant difference in responsible behavior between men and women in the study (*t* = −5.616, *p* < 0.001). Specifically, women exhibited higher levels of responsible behavior (*M* = 4.30, SD = 0.48) compared to men (*M* = 3.96, SD = 0.72). This finding suggests that gender may play a significant role in shaping environmental and cultural stewardship, potentially reflecting differences in societal roles or values.

#### 5.4.2 Age

ANOVA results indicated significant variations in responsible behavior across age groups (*F* = 20.937, *p* < 0.001). Younger respondents (aged 18–24, 25–34, and 35–44) demonstrated higher levels of responsible behavior compared to older age groups (45–54 and 55+). This pattern may reflect generational differences in environmental awareness or the influence of educational campaigns targeting younger populations.

#### 5.4.3 Educational level

Significant differences were also observed based on educational attainment (*F* = 19.697, *p* < 0.001). Respondents with postgraduate education, bachelor’s (or associate) degrees, and junior high school education scored higher than those with senior high school (or vocational) education. This finding underscores the importance of education in fostering environmental and cultural responsibility, potentially due to increased awareness and access to sustainability-related knowledge.

#### 5.4.4 Ethnicity

Ethnic background significantly influenced responsible behavior (*F* = 19.123, *p* < 0.001). Han and Tibetan respondents exhibited higher levels of responsible behavior compared to Yi and Qiang ethnic groups. These differences may stem from varying cultural traditions, values, or levels of engagement with tourism development initiatives.

#### 5.4.5 Duration of local residence

Significant differences were observed based on the length of residence (*F* = 38.941, *p* = 0.000). Respondents who had lived in the area for more than 30 years had the highest scores, followed by those who had lived there for 21–30 years. Respondents who had lived in the area for 6–10 years and 5 years or less had higher scores than those who had lived there for 11–20 years.

#### 5.4.6 Role in tourism development

Significant differences were found between respondents based on their involvement in the tourism industry (*F* = 44.910, *p* = 0.000). Tourism operators and managers had significantly higher scores than residents who were not directly involved in the tourism industry.

#### 5.4.7 Personal income and family income sources

Significant differences were observed based on personal annual tourism income (*F* = 26.392, *p* = 0.000). Respondents with an annual income of more than 50,000 RMB had higher scores than those with incomes between 10,000 and 30,000 RMB and between 5,000 and 10,000 RMB. Respondents with an income of less than 5,000 RMB had higher scores than those with incomes between 30,000 and 50,000 RMB. Those primarily dependent on tourism had the highest scores, followed by those with partial dependence, while those with minimal dependence scored higher than those not reliant on tourism at all (*F* = 14.046, *p* = 0.000).

## 6 Discussion, conclusion, and future directions

### 6.1 Discussion

This study integrates the theory of planned behavior, social capital theory, place attachment theory, and relative deprivation theory to construct a multidimensional framework for understanding individuals’ psycho-behavioral motivations in the context of tourism development, while accounting for complex factors such as social comparison and place attachment. Study results confirm that environmental and cultural attitude, government trust, and place attachment each have a significant positive impact on destination residents’ responsible behavior. The framework proves to be fully effective for identifying drivers of ethnic tourism area resident behavior.

While previous studies have primarily focused on the attitudes and behavior promoting environmental protection, we feel strongly that cultural protection is a similarly critical issue both for sustainable, high-quality tourism development and the future well-being of all people, but especially minority communities, such as are the focus of this study. Therefore, we integrated environmental and cultural attitudes into a single factor, ECA, and considered both environmentally and culturally responsible behaviors in defining the dependent variable, RB.

Our study reveals that ECA is resilient to relative deprivation, unlike government trust and place attachment. This resilience can be attributed to the intrinsic value that individuals place on their local environment and culture. Environmental and cultural attitudes are often deeply rooted in personal values and beliefs, which are less susceptible to external factors such as relative deprivation. In contrast, government trust and place attachment are more influenced by social comparisons and external conditions. The resilience of environmental and cultural attitudes to relative deprivation underscores the unique role of intrinsic values, while the moderation effects on trust and attachment highlight the fragility of extrinsic motivators—key insights enabled by our multidisciplinary framework. This finding suggests that ECA plays a unique role in promoting responsible behavior, independent of relative deprivation.

Study results reveal the strong multidimensional reach of environmental and cultural attitude in a specific cultural and touristic context. We found that ECA has a large and complex influence on destination resident behavior. This finding is consistent with those of Dunlap et al. (2000) and Tanner and Wölfing Kast (2003) on the positive predictive effect of environmental attitudes on environmental behavior, but additionally accounts for cultural attitudes and behavior, offering a new, integrated perspective for environmental protection and cultural policymaking at tourism destinations.

As asserted by social capital theory, trust is proven an important facilitator of community cohesion and collective action. Our study confirms that residents’ trust in authority is key to promoting their responsible behavior. We thus concur with Liu et al. (2014), but pinpoint more precisely how government trust translates into active support and participation in tourism development policy. This finding provides policymakers with a strategic direction for promoting community involvement and responsible behavior–by first raising levels of trust between government and locals.

This research provides new empirical support for place attachment theory and makes a strong case for policymakers to consider place attachment more seriously in the course of tourism development. The results confirm–as in Buta et al. (2014)–that local attachment to place exerts a positive influence on locally responsible behavior; however, our study goes a step further to explore this relationship by identifying the significant mediating effect of perceived tourism impact. Indeed, this study’s overall exploration of PTI’s mediating role deepens understanding of an otherwise difficult-to-grasp interplay between tourism development and destination resident psychology. This calls for more nuanced solutions and programs that attend to the ongoing effects of tourism in various areas of local life, and initial theoretical support for policies aimed at promoting environmental and cultural protection.

In addition, we investigated the moderating function of relative deprivation, a powerful and pervasive sensation with broad psychological repercussions. Our work echoes that of Wu et al. (2023), which similarly applies equity theory to environmental attitudes and behaviors. We found that a sense of relative deprivation may weaken the positive influence of government trust and place attachment on responsible behavior. This finding may help rationalize community residents’ attitudes and actions in the face of unevenly beneficial tourism development. Targeting the reduction of relative deprivation itself may be a winning strategy for strengthening government trust as well as place attachment, and ultimately promoting responsible behavior among locals.

The study also reveals the significant impact of factors such as gender, age, education level, ethnicity, length of residence, occupation, income, and family income sources on responsible behavior. These findings not only help us better understand the underlying driving mechanisms of residents’ responsible behavior but also provide important references for formulating relevant policies and educational programs. Specifically, gender is proven to be a crucial predictive factor, with females exhibiting higher levels of responsible behavior than males, potentially reflecting differences in societal gender roles and expectations. Age is also a notable factor, as young adults demonstrate greater commitment to environmental and cultural protection, likely due to their increased vitality and active participation in social causes. Education level is confirmed as a key determinant of responsible behavior, with individuals holding higher degrees and practical experience showing higher levels of responsible behavior, indicating that both knowledge and practical experience contribute to the promotion of environmental and cultural protection. Ethnicity also influences responsible behavior, with certain ethnic groups scoring higher, possibly reflecting their cultural traditions and values that prioritize harmony with nature and cultural preservation. Length of residence is positively correlated with responsible behavior, suggesting that long-term residents develop a stronger sense of belonging and responsibility towards their local environment and culture. Additionally, occupational roles, income levels, and reliance on tourism as a primary source of income are found to affect responsible behavior, with tourism operators, managers, and high-income individuals exhibiting greater responsibility, likely due to their professional duties and heightened awareness of conservation issues.

Overall, soundness of the model and data validate our integrated theoretical approach for this study’s context, while our results emphasize the importance of social comparison and individual psychological states in studies of environmental behavior. There are not only theoretical implications to be drawn here but also practical applications. To improve community participation and tourism sustainability, for example, one cannot ignore the complex interactions of cultural, social, emotional, and economic factors. Peng and Wang (2012) demonstrate how these factors are crucial to understanding the workings of relative deprivation, and our study clarifies how a sense of relative deprivation may catalyze active or passive resistance to various protective initiatives. Researchers and policymakers need this kind of broad yet precise understanding of destination residents to effectively grasp and manage tourism situations on the ground.

### 6.2 Conclusion

This study is an empirically based, in-depth exploration of ethnic-minority-area tourist destination residents’ culturally and environmentally responsible behavior and the internal drivers thereof. It finds that environmental and cultural attitude (ECA), government trust (GT), and place attachment (PA) respectively exert a significant positive effect on residents’ responsible behavior, and that perceived tourism impact (PTI) partially mediates each of these effects. On top of this, relative deprivation (RD) is found to significantly moderate GT and PA, but not ECA’s, effects on responsible behavior. As for a moderated mediating effect, RD is found to moderate PTI’s mediation of ECA and PA, but not GT’s, effects on responsible behavior.

These findings demonstrate the internal mechanisms of local psychological responses to tourism development, offering a new perspective on resident behavior and a theoretical foundation for improved environmental and cultural protection policy. Specifically, this study advances tourism sustainability research by integrating cultural responsibility into environmental frameworks, demonstrating the moderating role of relative deprivation, and providing a replicable model for balancing economic development with cultural-ecological preservation. Identifying the moderating effects of relative deprivation illuminates residents’ responses to tourism in view of their variable psychological states. For the immediate goal of promoting locally responsible behavior, our strategic targets should be government trust and place attachment, as improving these will lessen the counterproductive sense of relative deprivation. Understanding these chains of effect will be key to the creation of effective community engagement and resource management policies. Meanwhile, the moderated mediation effect identified in our study demonstrates how social psychology influences the relationships between perceived tourism impact and responsible behavior. Therefore, it may not be enough to improve residents’ perceptions of the changes brought by tourism; the perception of equitable benefit distribution may also need to be at a certain level to achieve highly responsible behavior and, ultimately, sustainable tourism development.

### 6.3 Management recommendations

Below we put forth seven sets of operationalizable governance and policy recommendations that should, through direct and indirect mechanisms, support locally responsible behavior among ethnic tourism destination residents. Furthermore, we believe that putting visible and earnest effort into each of these areas will generate government trust over and above the results of any specific initiative, compounding the benefits. It should also be noted, while this study primarily reveals the need to address socio-psychological dynamics (and relative deprivation) in fostering sustainable tourism development, there is an economic bedrock (and absolute deprivation) that must be kept in mind. Residents need not only positive attitudes and attachments, but a certain degree of support and empowerment to be able to turn away from extractive subsistence activities and join the tourism economy in the first place. Our recommendations reflect this comprehensive view and focus on win-win strategies for environmental and cultural sustainability, economic development, tourism product quality, and resident satisfaction.

#### 6.3.1 Strengthen community participation and environmental and cultural education

Knowledge is a foundational pillar for the development of environmental and cultural attitudes as well as place attachment. Education is also imperative if locals are to make informed decisions that are in the community’s best interests. Government should therefore cooperate with local schools to develop curricula that include relevant content on ecology, environment, and cultural heritage protection. That said, knowledge alone is insufficient; people also need guidance and opportunities to express or act in line with their environmental and cultural values. Government departments and tourism business partners can help by organizing cultural festivals and environmental events in the community, while using local media and public advertising to effectively generate publicity. To increase participation, they can design activities with experiential elements (e.g., ecological hiking tours led by indigenous elders or traditional handcraft workshops where residents teach tourists their techniques), and to address funding constraints, they can seek support from government or non-governmental organizations.

#### 6.3.2 Establish platforms for policy communication

As government trust has a direct effect on locals’ responsible behavior, resources invested in building trust will ultimately pay off as more successful sustainable development. In multiethnic districts, however, it can be difficult to develop trust across cultural and linguistic barriers. One thing government and tourism administrations can do is develop highly accessible platforms for transparent communication and online feedback in multiple languages. Media personnel can also receive specific training to ensure the precision and clarity of their messaging. This should reduce misunderstandings and help bridge the gap between policymakers and residents.

#### 6.3.3 Promote community involvement in decision-making

Locals will be more invested in the sustainability of the tourism and heritage economy if they have a say in its operation. Leaders are also only able to develop policy sensitive to local needs when they are in direct and regular contact with community members. Each administration should establish community advisory councils and put measures in place to guarantee a certain level of community participation and representativeness in tourism planning. They can also offer public capacity building workshops and seminars targeting relevant skills that residents may lack, improving their overall confidence and capability.

#### 6.3.4 Develop community-based tourism programs

Community-based tourism (CBT) provides an alternative to the mass-produced, locally nonspecific tourism experiences that distance visitors from local communities and often leave both parties dissatisfied. Building upon the above-described measures, governments should ultimately aim to partner with local tourism businesses to launch or expand ecotourism and cultural tourism projects informed by the unique characteristics of their locality. To get CBT up and running, they will need to provide micro-loans and entrepreneurial mentorships that support community members’ participation in tourism work. Meanwhile, to avoid conflicts between local culture and tourism programming, local governments should refine assessments of cultural appropriateness, encourage local innovation, and create spaces for multicultural expression.

#### 6.3.5 Optimize the benefit distribution system

One of the known problems in tourism development—and, as this study demonstrates, one felt deeply by destination residents—is the unequal distribution of benefits. Local governments should designate an equitable profit-sharing plan and ensure that community members can benefit directly from tourism profits. This could take the form of, for example, a community fund or bonus compensation system. They should also set up third-party oversight and improve transparency to reduce social inequality and the accompanying sense of deprivation.

#### 6.3.6 Protect and rejuvenate cultural heritage

As previously mentioned, cultural sustainability is too often put in third place behind economic and environmental sustainability. More deliberate, targeted efforts are needed to first understand and then protect the transmission of cultural heritage. Local governments can begin by conducting a heritage survey and use the findings to establish explicit plans for protection. They should actively support the transmission of arts and handcrafts and work with communities to organize cultural celebrations. They must find a better balance between the protection of cultural heritage and the needs of tourism to promote joint sustainable development.

#### 6.3.7 Establish a monitoring system for tourism impacts

To improve all-around decision-making, as well as the transparency and continuity of efforts, local governments should develop a continuous monitoring system to assess the environmental and social impacts of tourism, then regularly disseminate environmental and social impact reports. They can partner with research institutions to improve the professionalism of assessment techniques and adjust policy and administrative measures in light of assessment results. Even when confronted with technological limitations or resource constraints, administrations can make good use of existing technology and data collection methods on this front.

### 6.4 Current limitations and future prospects

Although this study offers preliminary results on resident behavior and tourism development of the Western Sichuan Ethnic Area, it has certain inevitable limitations, and owing to the importance and vulnerability of the region, there is urgent need to expand upon this research area with more extended and locally specific studies.

As for our research design, we cannot entirely rule out the possibility of confounding variables. Our study includes key predictors of RB such as environmental and cultural attitude, government trust, place attachment, and relative deprivation. However, other external factors—such as economic incentives, tourism dependence, or policy interventions—may be distorting the exact mechanisms of correlation. Future research should consider including control variables to account for these influences. For example, economic incentives and tourism dependence might directly affect residents’ willingness to engage in responsible behaviors, while policy interventions could alter the context in which these behaviors occur.

Another potential limitation lies in the integration of environmental and cultural attitudes in a single construct (ECA). On the one hand, this promotes the understanding of environment and culture as equally important and inextricably linked in the context of rural, place-based, ethnic tourism development. On the other hand, there are also benefits in separating the two dimensions. Future studies could isolate cultural factors from environmental factors to identify whether they operate differently in shaping responsible behavior. Research might reveal, for example, that cultural attitudes influence behavior through social norms and community values while environmental attitudes are more directly linked to specific conservation actions. Future studies might also consider including “social responsibility” in addition to cultural and environmental responsibility. We wanted to maintain close relevance to the local tourism context where culture/ethnicity and environment/place are both the products sold by and the identities held by community residents. However, including a broader social dimension may ultimately provide a more comprehensive understanding of responsible behavior in tourism contexts.

Finally, although bootstrapping provides a certain degree of robustness in mediation and moderation testing, there may yet be unidentified mediating or moderating variables at play. To delve more deeply, future research could investigate the roles of additional factors such as social norms, cultural values, or policy changes. Meanwhile, new factors shaping both behavior and policy will continue to appear with changes in technology, globalization, and climate. Future studies should consider which emergent variables are most significant for community development and assess their long-term impact.

## Data Availability

The raw data supporting the conclusions of this article will be made available by the authors, without undue reservation.
